# Invasion of the bucco-mandibular space by oral squamous cell carcinoma: histopathological analysis of invasion pattern

**DOI:** 10.3389/fonc.2023.1168376

**Published:** 2023-10-12

**Authors:** Takuma Kugimoto, Naoto Nishii, Yu Oikawa, Takeshi Kuroshima, Hideaki Hirai, Hirofumi Tomioka, Yasuyuki Michi, Kou Kayamori, Junichiro Sakamoto, Joe Iwanaga, R. Shane Tubbs, Tohru Ikeda, Masahiko Miura, Hiroyuki Harada

**Affiliations:** ^1^ Department of Oral and Maxillofacial Surgical Oncology, Division of Oral Health Science, Graduate School of Medical and Dental Sciences, Tokyo Medical and Dental University, Tokyo, Japan; ^2^ Department of Oral Pathology, Division of Oral Health Science, Graduate School of Medical and Dental Sciences, Tokyo Medical and Dental University, Tokyo, Japan; ^3^ Department of Dental Radiology and Radiation Oncology, Division of Oral Health Science, Graduate School of Medical and Dental Sciences, Tokyo Medical and Dental University, Tokyo, Japan; ^4^ Department of Oral and Maxillofacial Anatomy, Division of Oral Health Science, Graduate School of Medical and Dental Sciences, Tokyo Medical and Dental University, Tokyo, Japan; ^5^ Department of Neurosurgery, Tulane Center for Clinical Neurosciences, Tulane University School of Medicine, New Orleans, LA, United States

**Keywords:** bucco-mandibular space, oral squamous cell carcinoma, gingiva-buccal complex, mandible, buccinator muscle

## Abstract

**Background:**

This study aimed to determine the patterns of invasion of oral squamous cell carcinoma (OSCC) into the bucco-mandibular space (BMS) using detailed histopathological analysis and to assess clinical outcomes.

**Methods:**

Patients with OSCC who underwent segmental mandibulectomy or hemi-mandibulectomy combined with resection of the BMS between 2012 and 2021 were included. The invasions of the BMS were classified into three patterns. Pattern A was defined as a horizontal invasion, Pattern B as a vertical invasion, and Pattern C as an expansive invasion.

**Results:**

In total, 109 patients were reviewed. Of these 109 patients, the primary tumor affected the lower gingiva in 78 patients, the buccal mucosa in 18 patients, and was a primary intraosseous carcinoma of the mandible in 13 patients. Invasion of the BMS was significantly associated with a higher pathological T stage, positive/close margins, and lower disease-free survival (DFS) rates. The DFS rates were 86.7% and 66.0% in the BMS non-invasion and invasion groups, respectively. The DFS rates for each type of invasion were 82.1% for Pattern A, 67.4% for Pattern B, and 48.0% for Pattern C (P=0.277).

**Conclusion:**

Patients with BMS invasion have a poorer prognosis than those without invasion of the BMS. Therefore, adjuvant therapy is necessary, especially in Patterns B and C. Evaluation of preoperative BMS invasion patterns is important for predicting the prognosis of OSCC.

## 1 Introduction

**Figure 1:**
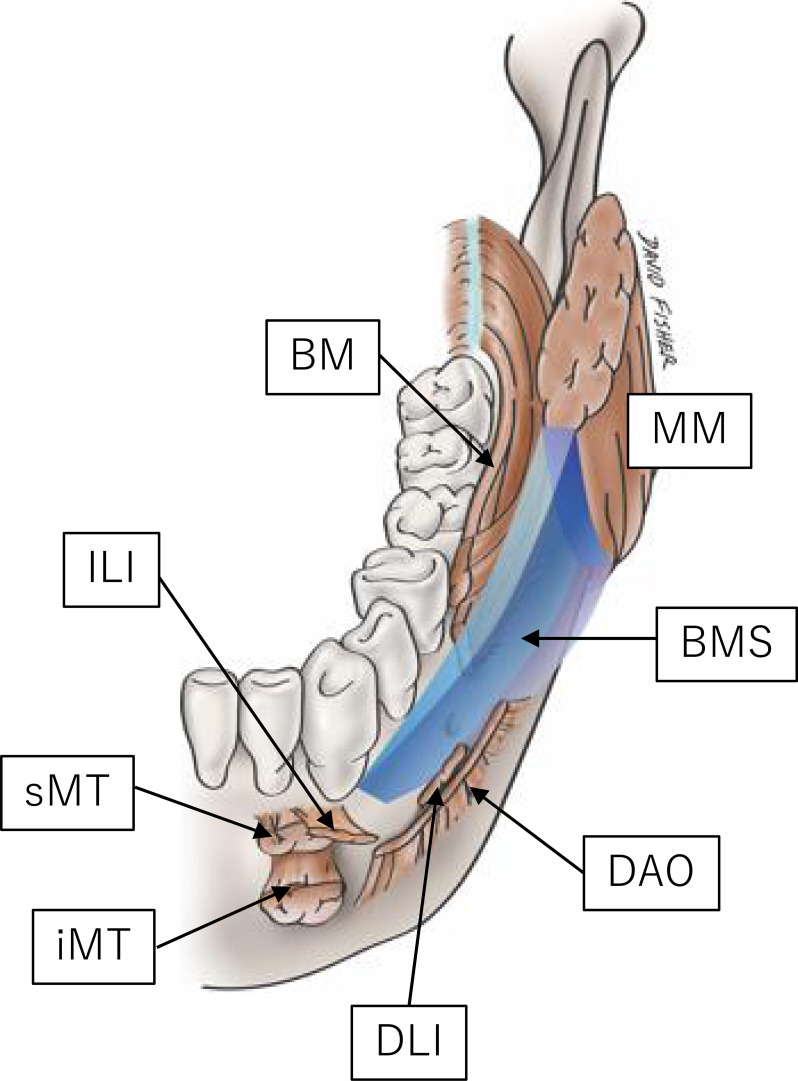
Graphic representation of the BMS (1). BM, buccinator; DAO, depressor anguli oris; DLI, depressor labii inferioris; ILI, incisivus labii inferioris; MM, masseter muscle; MT, (inferior and superior portion of the) mentalis. Reprinted from the figure in the article of (1) with permission.

The bucco-mandibular space (BMS) was described by Iwanaga et al. in 2017 as being located inferior to the buccal space and lateral to the mandible (1). This space is bounded by the incisivus labii inferioris and mentalis muscles anteriorly, anterior margin of the masseter muscle and its fascia posteriorly, depressor anguli oris inferiorly and laterally, by the lateral surface of the mandible medially, and by the platysma and its associated fascia, which is continuous with the masseteric fascia, superiorly (**Figure 1**). The fissure and loose connective tissues deep in the mucosa between the incisivus labii inferioris and buccinator muscles mark the entrance to the BMS. Oral squamous cell carcinoma (OSCC) arising in the gingivobuccal complex (GBC) might invade this space. The GBC includes the buccal mucosa, lower alveolus, gingivobuccal sulcus, and retromolar trigone (RMT). Several authors have emphasized the aggressive nature of this particular subsite, especially invasion of the mandible (2, 3). This study aimed to analyze the clinicopathological factors affecting survival outcomes in the patterns of OSCC invasion into the BMS. We hypothesized that there are differences in clinical outcomes due to different patterns of invasion of the BMS. The specific aims were to compare OSCC survival rates with or without BMS invasion and to examine whether the invasion pattern could be predicted preoperatively.

## 2 Materials and methods

### 2.1 Patients

Patients treated for OSCC in the Department of Oral and Maxillofacial Surgical Oncology at Tokyo Medical and Dental University between 2012 and 2021 were enrolled in this study. We examined all patients who were consecutively diagnosed with OSCC and underwent mandibulectomy at our hospital. Patients who met the following selection criteria were included: (i) diagnosed with OSCC and (ii) underwent segmental mandibulectomy or hemi-mandibulectomy combined with resection of the BMS. Patients with multiple OSCC and those who had other head and neck cancers were excluded from this study. In addition, patients with tumors in the anterior part of the mandible were excluded because the BMS is located in the mandibular molar region. Tumors were clinically staged using the Union International Cancer Control tumor-node-metastasis staging system (8th edition). Primary intraosseous carcinoma was classified as T4. For preoperative imaging categorization, we mainly used MR imaging (MRI). MR images were obtained using either a 1.5 T or 3 T MR machine using a standard extracranial head and neck protocol, which included T1- and T2-weighted, T2 fat suppression, and post-gadolinium (Gd) fat-suppressed T1-weighted sequences. Gd-enhanced T1-weighted coronal images with fat suppression were mainly assessed for the BMS invasion pattern. If contrast media were not available for specific reasons, the BMS invasion pattern was evaluated on T2-weighted images. Following decalcification, each specimen was sliced vertically from the anterior to the posterior at 1-cm intervals. Radiological and pathological assessments of the BMS invasion pattern were performed at the maximum cross-section. The BMS invasion pattern was evaluated in slices containing the deepest part of BMS invasion in the coronal plane. Pathological slides were reviewed to determine the extent of tumor invasion into the BMS. Postoperative chemotherapy and/or radiotherapy (2.0 Gy/fraction, total 50–66 Gy) was administered to patients with close or positive margins of the primary tumor or ≥4 histological cervical lymph node metastases and those positive for extranodal extension with platinum-based anticancer agents administered concurrently, if possible (4). Elderly patients and patients with renal dysfunction were administered S-1. This study was conducted in accordance with the Declaration of Helsinki and was approved by the ethics committee of Tokyo Medical and Dental University, Faculty of Dentistry (No. D2015-600). Notices about automatic opt-in consent for the study and method for opting-out were posted in the hospital, as approved by the Ethics Committee of the university. Participants were informed that there was an option for an opt-out of this retrospective research at any time.

### 2.2 Radiological and pathological evaluations of the bucco-mandibular space invasion pattern

**Figure 2:**
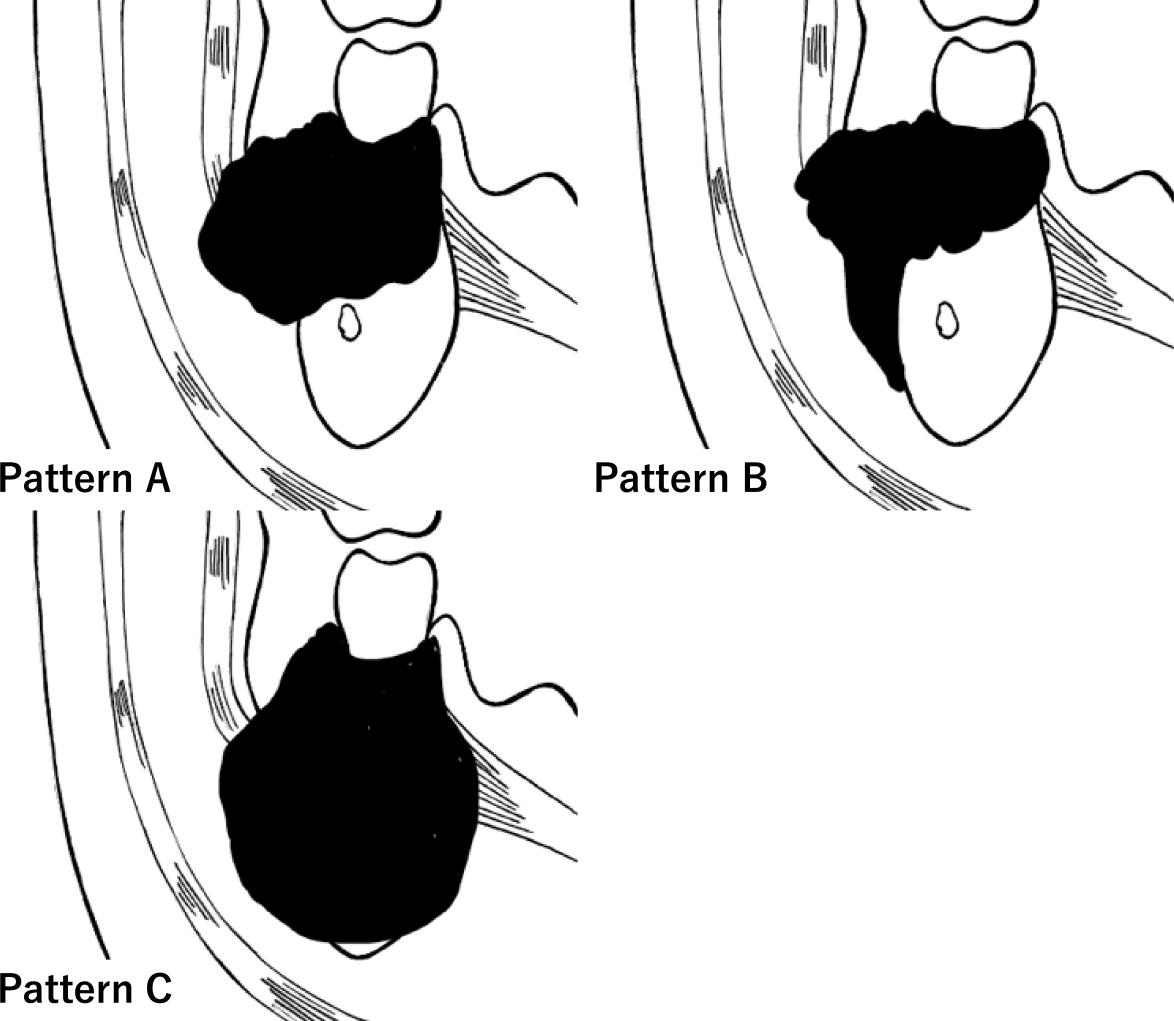
Graphic representation of the clinical classification of pattern of the bucco-mandibular space (BMS) invasion. Invasion of oral squamous cell carcinoma into the BMS (arrow).

**Figure 3:**
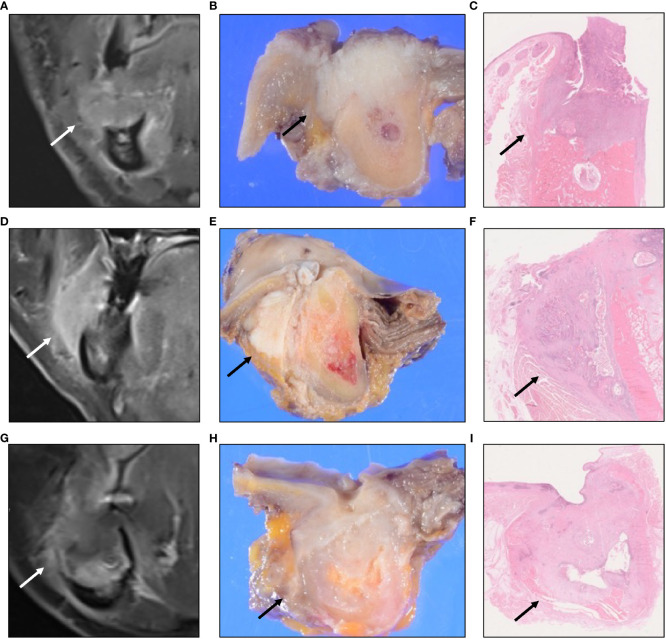
Pattern A (horizontal type) is an infiltration type that invades to the bucco-mandibular space (BMS) while compressing **(A–C)**. Pattern B (vertical type) is an infiltration type that invades to the BMS along the periosteum **(D–F)**. Pattern C (expansive type) is an infiltration type in which a tumor replaces the mandible and invades the BMS while expanding **(G–I)**. Coronal gadolinium-enhanced T1-weighted magnetic resonance imaging **(A, D, G)**. Macroscopic findings show the cut surface **(B, E, H)**. Histological findings **(C, F, I)**.

We developed an original classification system based on the pattern of tumor invasion, which was categorized according to its spread into the BMS. According to this method, invasion of the BMS can be classified based on its “pattern of invasion” into three groups: A, B, and C (**Figure 2**). Pattern A (horizontal type) is an infiltration type that invades the BMS during compression (**Figure 3**). Pattern B (vertical type) is an infiltration type that invades the BMS along the periosteum (**Figure 3**). Pattern C (expansive type) is an infiltration type in which the tumor replaces the mandible and invades the BMS while expanding (**Figure 3**). For radiological evaluation, All MR images were evaluated independently by an oral surgeon (T Kugimoto) and an expert radiologist (JS) who were blinded to the case history and outcome. Furthermore, JS re-reviewed after an interval of at least two weeks. At the second observation, JS was unaware of the previous evaluation. For pathological evaluation, all pathological slides were evaluated independently by T Kugimoto and an expert pathologist (KK) who were blinded to the case history and outcome. Furthermore, KK re-reviewed after an interval of at least two weeks. At the second observation, KK was unaware of the previous evaluation.

### 2.3 Statistical analyses

Comparisons between categorical variables were performed using the chi-squared or Fisher’s exact test, as appropriate. Sensitivity, specificity, positive predictive value (PPV), negative predictive value (NPV), and accuracy were calculated for the BMS non-invasion group versus the BMS invasion group for the overall cohort and for each invasion pattern. Survival (disease-specific survival and disease-free survival [DFS]) were estimated using the Kaplan–Meier method, and groups were compared using the log-rank test. Inter- and intra-rater reliability was calculated using Cohen’s kappa (< 0.00 no agreement; 0.00-0.20 slight agreement; 0.21-0.40 fair agreement; 0.41-0.60 moderate agreement; 0.61-0.80 substantial agreement; > 0.80 almost perfect agreement). All statistical analyses were performed using R-3.5.3 statistical software (https://www.r-project.org/). Statistical significance was set at P<0.05.

## 3 Results

### 3.1 Patient characteristics

The patient characteristics are summarized in **Table 1**. In total, 109 patients met the inclusion criteria: 63 and 46 in the BMS non-invasion and invasion groups, respectively. A total of 47.6% and 97.8% of the patients had pT3 to 4 stage in the BMS non-invasion and invasion groups, respectively (P < 0.01). Neck dissection was performed in all patients. A total of 24 (38.1%) patients in the BMS non-invasion group showed positive nodes (pN1 [n=10] and pN2 to 3 [n=14]), whereas 24 (52.2%) patients in the BMS invasion group showed positive nodes (pN1 [n=7] and pN2 to 3 [n=17]) (P=0.39). In the BMS invasion group, 16 patients presented with Pattern A, 19 presented with Pattern B, and 11 presented with Pattern C. Skin involvement was observed in six patients (Pattern A=2, Pattern B=3, and Pattern C=1). Postoperative chemoradiotherapy was administered to 19 (41.3%) patients in the BMS invasion group and 9 (14.3%) patients in the BMS non-invasion group. In the BMS invasion group, concomitant anticancer drugs used during postoperative radiotherapy were cisplatin (CDDP) in 9 patients and S-1 in 10 patients. In the BMS non-invasion group, concomitant anticancer drugs used during postoperative radiotherapy were CDDP in 4 patients, S-1 in 5 patients.

**Table 1:** Patient characteristics.

Variable		BMS non-invasion group (n=63) (%)	BMS invasion group (n=46) (%)	P-value
Age	<60	12 (19.0)	13 (28.3)	0.37
	>60	51 (81.0)	33 (71.7)	
Sex	Male	39 (61.9)	29 (63.0)	1
	Female	24 (38.1)	17 (37.0)	
Primary	Lower gingiva	44 (69.8)	34 (73.9)	0.01
	Buccal mucosa	15 (23.8)	3 (6.5)	
	Primary intraosseous carcinoma of mandible	4 (6.4)	9 (19.6)	
Differentiation	Well	27 (42.9)	19 (41.3)	0.52
	Moderately	33 (52.4)	22 (47.8)	
	Poorly	3 (6.5)	5 (10.9)	
Mandibulectomy	Segmental	56 (88.9)	36 (78.3)	0.21
	Hemi-mandibulectomy	7 (11.1)	10 (21.7)	
pT	T1	7 (11.1)	0 (0.0)	<0.01
	T2	26 (41.3)	1 (2.2)	
	T3	4 (6.3)	6 (13.0)	
	T4	26 (41.3)	39 (84.8)	
pN	N0	39 (61.9)	22 (47.8)	0.39
	N1	10 (15.9)	7 (15.2)	
	N2	7 (11.1)	8 (17.4)	
	N3	7 (11.1)	9 (19.6)	
Horizontal margin	Negative	55 (87.3)	38 (82.6)	0.68
	Close/positive	8 (12.7)	8 (17.4)	
Vertical margin	Negative	50 (79.4)	16 (34.8)	<0.01
	Close/positive	13 (20.6)	30 (65.2)	
Postoperative chemoradiotherapy	Absent	54 (85.7)	27 (58.7)	<0.01
	Present	9 (14.3)	19 (41.3)	

### 3.2 Radiological and pathological evaluations of the BMS invasion pattern analysis

Inter- and intra-rater reliability for radiological evaluation demonstrated almost good agreement (κ=0.70 and κ=0.83, respectively). Inter- and intra-rater reliability for pathological evaluation demonstrated almost perfect agreement (κ=0.83 and κ=0.93, respectively). MRI findings were reviewed in 102 of the 109 patients (**Table 2**). Seven patients could not be evaluated by MRI because they were claustrophobic, or they had metals in their body. Evaluation of the BMS invasion by MRI had a sensitivity of 100%, specificity of 84.2%, PPV of 83.3%, NPV of 100%, and accuracy of 91.2%. Preoperative MRI correctly predicted the BMS invasion pattern after pathological analysis in 68.9% of the patients in the BMS invasion group. With respect to Patterns A (n=16), B (n=19), and C (n=11), there was agreement in 11 (68.8%), 10 (55.6%), and 10 (90.9%) patients, respectively. Two cases were evaluated as Pattern C in the radiological classification owing to inflammatory spread on incisional biopsy despite the pathological classification as Pattern A.

**Table 2:** Magnetic resonance imaging and pathological evaluation of the BMS invasion pattern.

		Pathological classification				Total
		Non-invasion*	Pattern A	Pattern B^†^	Pattern C	
Radiological classification	Non-invasion	48	0	0	0	48
	Pattern A	4	11	2	1	18
	Pattern B	4	3	10	0	17
	Pattern C	1	2	6	10	19
Total		57	16	18	11	102

BMS, bucco-mandibular space.

* Six patients were excluded from this study.

^†^ One patient was excluded from this study.

### 3.3 Histopathological analysis by the BMS invasion pattern

Histopathological analysis showed that the BMS invasion group had a higher pathological T stage and higher frequency of pathological cervical lymph node metastasis than the BMS non-invasion group (**Table 1**). The relationships between invasion patterns and YK classification (5) and Grade classification (WHO proposal) were analyzed, but no correlations were found. Among the BMS invasion group, 10 patients were of pT4b (Pattern A=0, Pattern B=5, and Pattern C=5). Comparing the horizontal and vertical margins of the BMS non-invasion and invasion groups, there was no significant difference in the horizontal margin (P=0.68), but there was a significant difference in the vertical margin (P<0.01) (**Table 1**). In the BMS invasion group, it was difficult to secure a sufficiently deep safety margin in the vertical margin, especially in Patterns B and C (**Table 3**). In Patterns B and C, the tumor had spread submucosally into the BMS.

**Table 3:** Pathological tumor-node classification and margin status of the bucco-mandibular space invasion pattern.

		Pattern A (n=16) (%)	Pattern B (n=19) (%)	Pattern C (n=11) (%)
Primary	Lower gingiva	14 (87.5)	13 (68.4)	7 (63.6)
	Buccal mucosa	1 (6.3)	2 (10.5)	0 (0.0)
	Primary intraosseous carcinoma of mandible	1 (6.3)	4 (21.1)	4 (36.4)
pT	pT1–2	1 (6.3)	0 (0.0)	0 (0.0)
	pT3–4	15 (93.7)	19 (100)	11 (100)
pN	pN0	10 (62.5)	8 (42.1)	4 (36.4)
	pN1	1 (6.3)	5 (26.3)	1 (9.1)
	pN2–3	5 (31.2)	6 (31.6)	6 (54.5)
Horizontal margin	Positive	0 (0.0)	1 (5.3)	1 (9.1)
	Close	1 (6.3)	2 (10.5)	3 (27.3)
	Negative	15 (93.7)	16 (84.2)	7 (63.6)
Vertical margin	Positive	0 (0.0)	0 (0.0)	1 (9.1)
	Close	9 (56.3)	13 (68.4)	7 (63.6)
	Negative	7 (43.7)	6 (31.6)	3 (27.3)

### 3.4 Treatment outcomes

**Figure 4:**
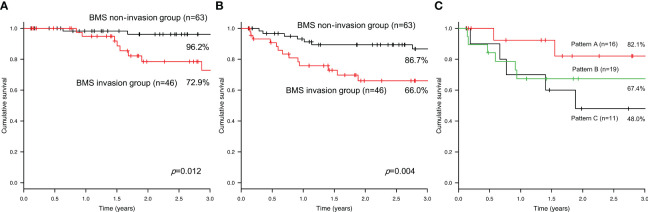
The 3-year disease-specific survival **(A)** and disease-free survival **(B)** rates in the bucco-mandibular space non-invasion and invasion groups. The 3-year disease-free survival rate in each pattern of the bucco-mandibular space invasion group **(C)**.

In the BMS non-invasion group, seven patients developed recurrence, specifically local recurrence in three patients, regional recurrence in three patients, and distant metastasis in one patient. In the BMS invasion group, 14 patients developed recurrence, specifically local recurrence in five patients, regional recurrence in two patients, and distant metastasis in seven patients. The 3-year disease-specific survival rates were 96.2% and 72.9% in the BMS non-invasion and invasion groups, respectively (**Figure 4**). There was a significant difference between the two groups (P=0.012). The 3-year DFS rates were 86.7% and 66.0% in the BMS non-invasion and invasion groups, respectively (**Figure 4**). A significant difference was observed between the two groups (P=0.004). In the BMS invasion group, the 3-year DFS rates were 82.1%, 67.4%, and 48.0% in Patterns A, B, and C, respectively (P=0.277) (**Figure 4**).

## 4 Discussion

This study confirmed the oncological significance of the BMS invasion pattern for predicting DFS in patients with OSCC, indicating that Patterns B and C were adverse prognostic factors for OSCC. We present a new system for classifying BMS invasion patterns that is simple, easy to learn, and clinically significant. This pattern-based classification system better predicts the risk of recurrence and stratifies patients into three distinct groups.

**Figure 5:**
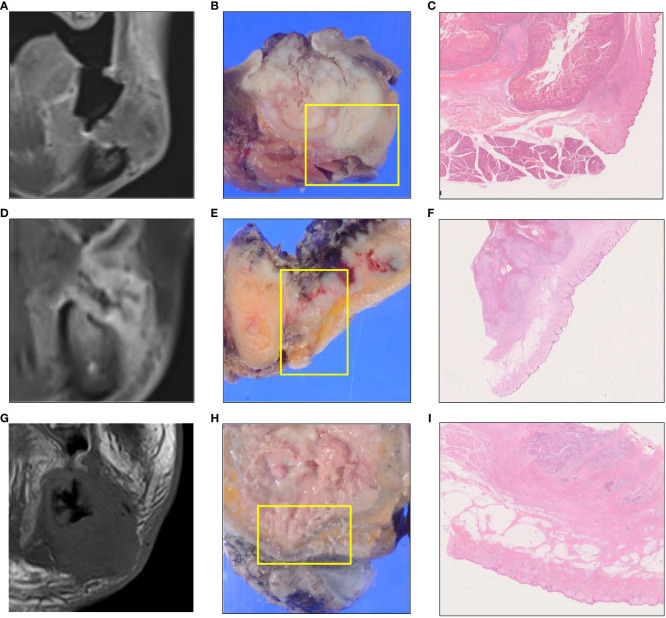
MR and pathological images showing the tumor skin infiltration or the tumor region closest to the skin for each invasion pattern. A skin infiltration case of Pattern A. **(A–C)**. A skin infiltration case of Pattern B. **(D–F)**. A skin infiltration case of Pattern C. **(G–I)**. Coronal gadolinium-enhanced T1-weighted magnetic resonance imaging **(A, D, G)**. Macroscopic findings show the cut surface **(B, E, H)**. Histological findings **(C, F, I)**.

Iwanaga et al. reported that fresh cadavers revealed a specimen with a gap between the depressor anguli oris and platysma muscles (1). The BMS invasion of OSCC is considered almost synonymous with soft tissue invasion of GBC carcinoma. GBC carcinoma with skin involvement shows poor survival outcomes (6, 7). In Patterns B and C, the tumor had spread submucosally into the BMS, which was considered to be the pathway for skin invasion (**Figure 5**).

Ota et al. examined the depth of tumor invasion in relation to the anatomical layer of the cheek wall and developed a classification system for tumor invasion (8). The buccinator muscle has been suggested as a possible barrier to tumor invasion. However, as mentioned above, there is a fissure between the lateral border of the incisivus labii inferioris and the anterior border of the buccinator muscle, which cannot be applied to all GBC carcinomas. The GBC extends along the surface mucosa, and the submucosal soft tissue approaches the buccal or labial gingiva. From this point onward, the tumor does not extend directly through the intact periosteum and cortical bone toward the cancellous part because the periosteum acts as a significant protective barrier (9).

Imaging in GBC carcinoma is crucial for evaluating soft tissue spread and bone involvement (10). The accuracy of different imaging techniques for assessing the BMS invasion in OSCC remains controversial. Additionally, several comparative studies have identified MRI as the preferred method for the detection of BMS invasion. However, there were cases in which it was difficult to evaluate the BMS invasion pattern and mandibular invasion using MRI after incisional biopsy. Studies on oral cancer have overestimated the thickness of the tumor after biopsy and attributed it to high signal intensity hemorrhage and edema (11–13). In fact, two cases were evaluated as Pattern C in the radiological classification owing to inflammatory spread on incisional biopsy despite the pathological classification as Pattern A. Although MR may be more sensitive in detecting BMS abnormalities, it may be limited because of susceptibility artifacts from the dental material.

In gingivobuccal cancer, large tumors with paramandibular disease are likely to have multiple routes of entry, which contraindicates mandibular conservation. Deeply invading tumors of the soft tissues enter the jaw at both the alveolus and lower border, risking a compromised margin in conservative resections of the mandible (14). Oncological safety can be achieved by positioning the bone cut margin corresponding to the adjacent soft tissue cut margins in segmental mandibulectomy. Histopathological analysis of GBC carcinoma shows patterns of invasion and routes of tumor entry into the mandible, indicating that the length of cortical invasion corresponds to adjacent soft tissue involvement and does not indicate mandibular invasion beyond soft tissue involvement (2). Histopathological analysis showed that GBC had a higher pathological T stage and positive margins (15). Among our BMS invasion cases, there were 10 cases of pT4b. Because of their close proximity to the RMT region, GBC carcinoma tends to invade the RMT at an early stage and hence can spread through the RMT into multiple compartments including the masticator space (16–18). Walvekar et al. recommended aggressive surgical therapy for advanced-stage cancers of the GBC, which includes a wide three-dimensional resection to account for soft tissue and bony infiltrations and adjuvant therapy in the presence of adverse features as salvage rates for recurrent tumors are poor (19). This correlates with the aggressive nature of GBC carcinoma and is further validated by intraoperative observations that acquiring negative deep soft tissue margins in a circumferential manner is the most challenging part of these resections (20). Min et al. reported an association between muscle invasion and cervical lymph node metastasis in OSCC of the posterior mandibular alveolar ridge (21). In this study, more than 50% of patients with Patterns B and C had metastatic lymph nodes and required aggressive treatment.

The pattern of soft tissue invasion has become a useful tool for further characterizing the biological behavior of OSCC. The worst aggressive pattern of invasion in the surrounding soft tissue has been previously shown to be predictive of higher local recurrence and poorer survival (22). The worst aggressive pattern of invasion in OSCC tumors exhibited a trend of more frequent mandibular invasion and an infiltrative pattern of invasion (3). Another study performed immunohistochemistry to identify biomarkers significantly associated with histological adipose tissue invasion in OSCC (23).

There are some limitations in our study that should be acknowledged. First, this study was its retrospective nature. Second, the border between the BMS and buccal space is unclear, especially in MRI and pathological tissue. The buccinator muscle originates from the pterygomandibular raphe and buccinator crest, and the buccal space exists lateral to the buccinator muscle. If a tumor originates from the molar region, the initial lateral infiltration may be difficult to distinguish between the buccal space invasion and the BMS invasion. Further studies are required to verify this new mechanism and clarify the relationship between soft tissue and bone patterns of invasion.

## 5 Conclusion

Patients with BMS invasion have poorer prognosis than those without BMS invasion. Therefore, adjuvant therapy is necessary, especially in Patterns B and C. Evaluation of preoperative BMS invasion patterns is important for predicting the prognosis of OSCC.

## Data availability statement

The original contributions presented in the study are included in the article/supplementary material. Further inquiries can be directed to the corresponding author.

## Ethics statement

This study was conducted in accordance with the Declaration of Helsinki and was approved by the ethics committee of Tokyo Medical and Dental University, Faculty of Dentistry (No. D2015-600). Notices about automatic opt-in consent for the study and method for opting-out were posted in the hospital, as approved by the Ethics Committee of the university. Participants were informed that there was an option for an opt-out of this retrospective research at any time. The study was conducted in accordance with the local legislation and institutional requirements.

## Author contributions

TKug, and HHa conceptualized this study. TKug, NN, YO, TKur, HHi, HT, and YM curated and investigated the data. TKug conducted the formal analysis. KK and TI performed the pathological investigation. JS and MM performed the radiological investigation. JI and RST conducted an anatomical consultation. TKug wrote the draft of the manuscript, and TKug and HHa reviewed and edited the manuscript. All authors contributed to the article and approved the submitted version.
